# Safety and Efficacy of Double-Layer Pancreaticojejunostomy With a Wide Jejunal Opening: Initial Experience From a Consecutive Case Series

**DOI:** 10.7759/cureus.105943

**Published:** 2026-03-26

**Authors:** Kaushal Yadav

**Affiliations:** 1 Surgical Oncology, Max Hospital, Gurgaon, Gurgaon, IND

**Keywords:** anastomosis leak, carcinoma pancreas, pancreatic duct, pancreaticoduodenectomy, pancreaticojejunostomy, postoperative pancreatic fistula

## Abstract

Introduction: Postoperative pancreatic fistula (POPF) remains the most significant complication following pancreaticoduodenectomy. The duct-to-mucosa (DTM) technique has gained attention for its potential to reduce POPF rates. While most published techniques involve a small jejunal opening (1 cm), this study evaluates the safety and feasibility of wide jejunal opening (approximately 4 cm) anastomosis in a consecutive case series.

Method: This retrospective analysis included 12 consecutive patients who underwent pancreaticoduodenectomy with double-layer DTM pancreaticojejunostomy using a wide jejunal opening at our institution, between January 2017 and August 2024. Outcome related to clinically relevant postoperative pancreatic fistula (CR-POPF), drain fluid amylase levels, delayed gastric emptying (DGE), operative time, hospital stay, and complications were analyzed.

Results: The mean patient age was 59.8 ± 10.5 years, with a median pancreatic duct diameter of 3.5 mm (range: 2-6 mm). The mean anastomosis time was 51.25 ± 7.11 (range: 40-60) minutes. No patient developed CR-POPF. DGE occurred in 10 patients (83.3%), predominantly grade C in seven (58.3%). Overall morbidity was 41.7% (5/12).

Conclusion: Although this study has a limited number of patients, it emphasizes the safety of double-layer DTM pancreaticojejunostomy with a wide jejunal opening. The technique's complexity and longer anastomosis time must be balanced against potential benefits in challenging scenarios such as small duct diameter or soft pancreas.

## Introduction

Pancreaticoduodenectomy (PD), first described by Whipple in 1935, remains the standard curative treatment for periampullary malignancies, including pancreatic head, ampullary, distal bile duct, and duodenal cancers. Despite significant advances in surgical technique, anesthesia, and perioperative care, PD continues to be associated with substantial morbidity, with reported complication rates of 30%-60% even in high-volume centers [1]. Postoperative pancreatic fistula (POPF) remains the most feared complication following PD.

Over the past four decades, numerous pancreaticojejunostomy (PJ) techniques have been developed to minimize POPF risk. The two principal approaches are invagination (dunking) and duct-to-mucosa (DTM) anastomosis. In the invagination technique, the pancreatic remnant is telescoped into the jejunal lumen and secured with seromuscular sutures. The DTM technique involves direct mucosa-to-mucosa anastomosis between the pancreatic duct and jejunal mucosa, typically reinforced with an outer layer of seromuscular sutures between the pancreatic capsule and jejunal serosa [2,3]. This technique allows for precise control and minimizes tension on the anastomosis [4,5]. The DTM technique has demonstrated promising results in reducing POPF rates. Studies have shown that the incidence of clinically relevant POPF (CR-POPF) with this method ranges between 5% and 15%, which is comparable or better than other techniques such as invagination PJ [6-8]. A meta-analysis of randomized controlled trials found that DTM anastomosis significantly reduced the incidence of grade B/C POPF compared to invagination techniques, particularly in patients with a soft pancreas [9,10]. Studies have reported low mortality rates (0.7%-4.8%) following DTM anastomosis, further highlighting its safety and efficacy [11,12]. Multiple modifications of these basic techniques have been described, including the Blumgart anastomosis (which uses transpancreatic U-sutures), the Heidelberg technique (standardized DTM with specific suture configuration), and the Kakita technique (binding PJ).

An often-overlooked technical variable in PJ is the size of the jejunal opening. In this study, PJ with a wide jejunal opening implies that the opening in the Roux limb of the jejunum is equivalent or slightly shorter in diameter to the pancreas remnant (approximately 4 cm). PJ with a small jejunal opening is equivalent in size to the pancreatic duct (1 cm). Studies have documented that DTM has favorable outcomes and is safe with reduced rates of clinically relevant POPF [13,14]. A single-center small randomized trial comparing large versus small jejunal incisions found that smaller incisions resulted in a lower incidence of POPF (4% vs. 13.6%) and postpancreatectomy hemorrhage (PPH) (8% vs. 36%) [15]. The authors suggested a "pancreatic duct-oriented" approach: if the duct is large, a small jejunal incision should be made, and if the duct is less than 3 mm, a larger jejunal incision should be preferred for PJ [15]. A study comparing four-layer wide jejunal opening technique with direct mucosa-to-mucosa anastomosis demonstrated a lower rate of POPF (5.6%) compared to traditional methods (17%) [16]. Despite the theoretical advantages of the wide jejunal opening technique, particularly in patients with challenging pancreatic anatomy with small duct, soft pancreas, and unidentified accessory duct, comparative data on jejunal opening size are scarce [17]. The primary objective of this study is to assess the safety and feasibility of double-layer DTM PJ with a wide jejunal opening in consecutive patients undergoing PD. Secondary objectives included evaluation of anastomosis time, bleeding, hospital stay, delayed gastric emptying (DGE) rates, and overall perioperative outcomes.

## Materials and methods

This retrospective observational case series includes 12 consecutive patients who underwent PD with double-layer DTM PJ using a wide jejunal opening at our institution, between January 2017 and August 2024. Given the retrospective nature of the study and use of de-identified data, the requirement for individual informed consent was waived by the ethics committee. All adult patients (≥18 years) who underwent PD with PJ for periampullary malignancy were included. Patients treated with total pancreatectomy, pancreaticogastrostomy, and those with incomplete medical records or who were lost to follow-up before six months were excluded from the study.

Intraoperative variables of pancreatic duct diameter and texture were noted as per the International Study Group of Pancreatic Surgery (ISGPS) classification [18]. Primary outcome of CR-POPF was reported as per the ISGPS 2016 updates [19]. Operative time and blood loss were noted, and the drain fluid amylase was documented on postoperative day (POD) three and POD five. Biochemical parameters, aside from the complete blood count, like C-reactive protein and serum procalcitonin, were not reported consistently and were only conducted when necessary. Similarly, radiological imaging was reserved for cases where there was a clinical suspicion. Postsurgical complications were recorded as per the Clavien-Dindo classification [20]. The secondary outcome of DGE was recorded as per the ISGPS definition [21]. Preoperative biliary drainage (PBD) for obstructive jaundice and cholangitis was documented, and the duration for postoperative drain removal was analyzed.

Surgical technique

**Figure 1:**
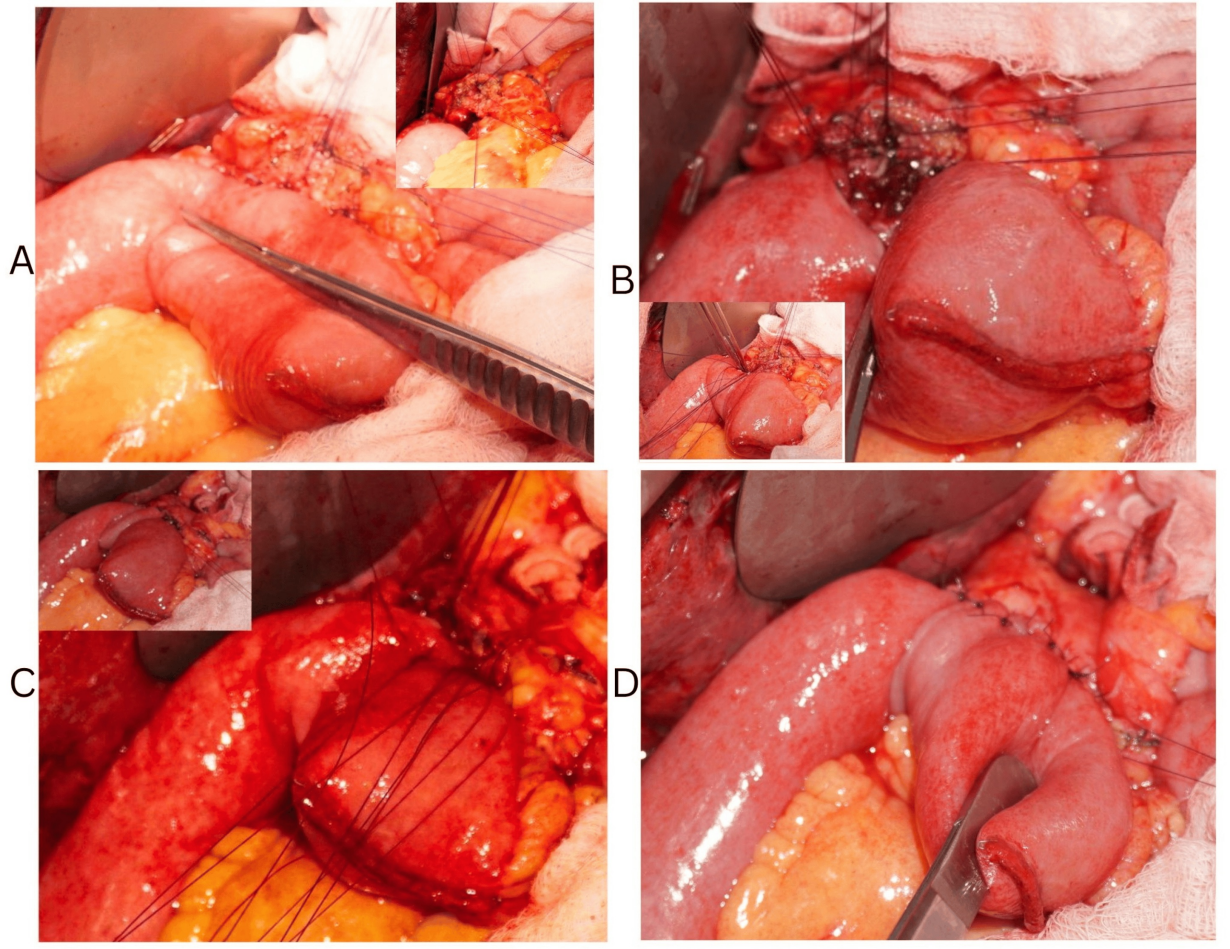
Pancreatojejunostomy with a wide jejunal opening. (A) Posterior outer layer; inset showing ductal sutures. (B) Posterior inner layer. (C) Anterior inner layer. (D) Anterior outer layer.

Standard or pylorus-preserving PD was performed based on tumor location and extent. The pancreas was transected at the neck over the superior mesenteric-portal vein confluence. Pancreatic texture was assessed by digital palpation and classified as soft (easily indented) or firm (resistant to indentation). A retrocolic jejunum loop is brought into the subhepatic location through a mesocolic window to the right of the middle colic vessels. The pancreatic duct is identified, and a ductal stent is placed. We routinely give octreotide 100 mcg one hour before pancreatic transection. Two or three polydioxanone (PDS) 4-0 or 5-0 sutures are placed first on the posterior side of the main pancreatic duct (MPD) in an inside-out fashion. Again, two or three sutures are placed on the anterior side of the MPD in an outside-in fashion. Respective sutures are held in mosquito forceps and placed on the left side of the body while taking care to identify each suture separately by covering them with a gauze piece (Figure 1A).

First (Posterior-Outer) Layer

The posterior-most (first) layer of suture is started by inserting sutures over the posterior surface of the pancreas remnant and coming out through the seromuscular layer of the jejunum. All sutures are inserted first in an interrupted fashion and tied later by parachuting jejunum over the posterior surface of the pancreas. Care is taken to place the notes over the surface of the jejunum rather than the pancreas. After tying all sutures, they are cut. This first layer will invaginate approximately 1 cm of the pancreas when the jejunum is incised widely next (Figure 1A).

Second (Posterior-Inner) Layer

An incision is made in the jejunum on the antimesenteric border with a size equal to or minutely less than the size of the pancreatic remnant diameter. In the second (posterior inner) layer, each suture begins by inserting into the posterior half of the pancreatic parenchyma, then exits through the posterior pancreatic surface, and finally inserts into the jejunal serosa before piercing the mucosa and entering the jejunal opening. These posterior inner-layer sutures start at the cephalad end and proceed caudally one by one. While previously placed posterior duct sutures are encountered, they are incorporated in this layer. Inside-out placed posterior duct sutures are now inserted from the serosa to the inside of the jejunum sequentially. After placing duct sutures, the posterior pancreatic parenchymal sutures are continued caudally in a manner consistent with the cranial ones. While placing these posterior ductal sutures, adequate duct-to-jejunal mucosa coaptation was ensured. With posterior parenchymal sutures, the pancreas was adequately approximated with the jejunal mucosa, both cranial and caudal to the pancreatic duct. After placing all sutures meticulously, they are tied sequentially and cut afterwards (Figure 1B).

Third (Anterior-Inner) Layer

The anterior inner layer of sutures is placed similarly in an outside-in fashion. The suture is inserted over the anterior pancreatic surface, then exits through the anterior half of the pancreatic parenchyma to go inside the anterior jejunal opening, piercing the mucosa, and finally comes out through the anterior jejunal serosa. Previously placed anterior ductal sutures are incorporated to complete this anterior inner layer. All sutures are tied sequentially and then cut (Figure 1C).

Fourth (Anterior-Outer) Layer

**Figure 2:**
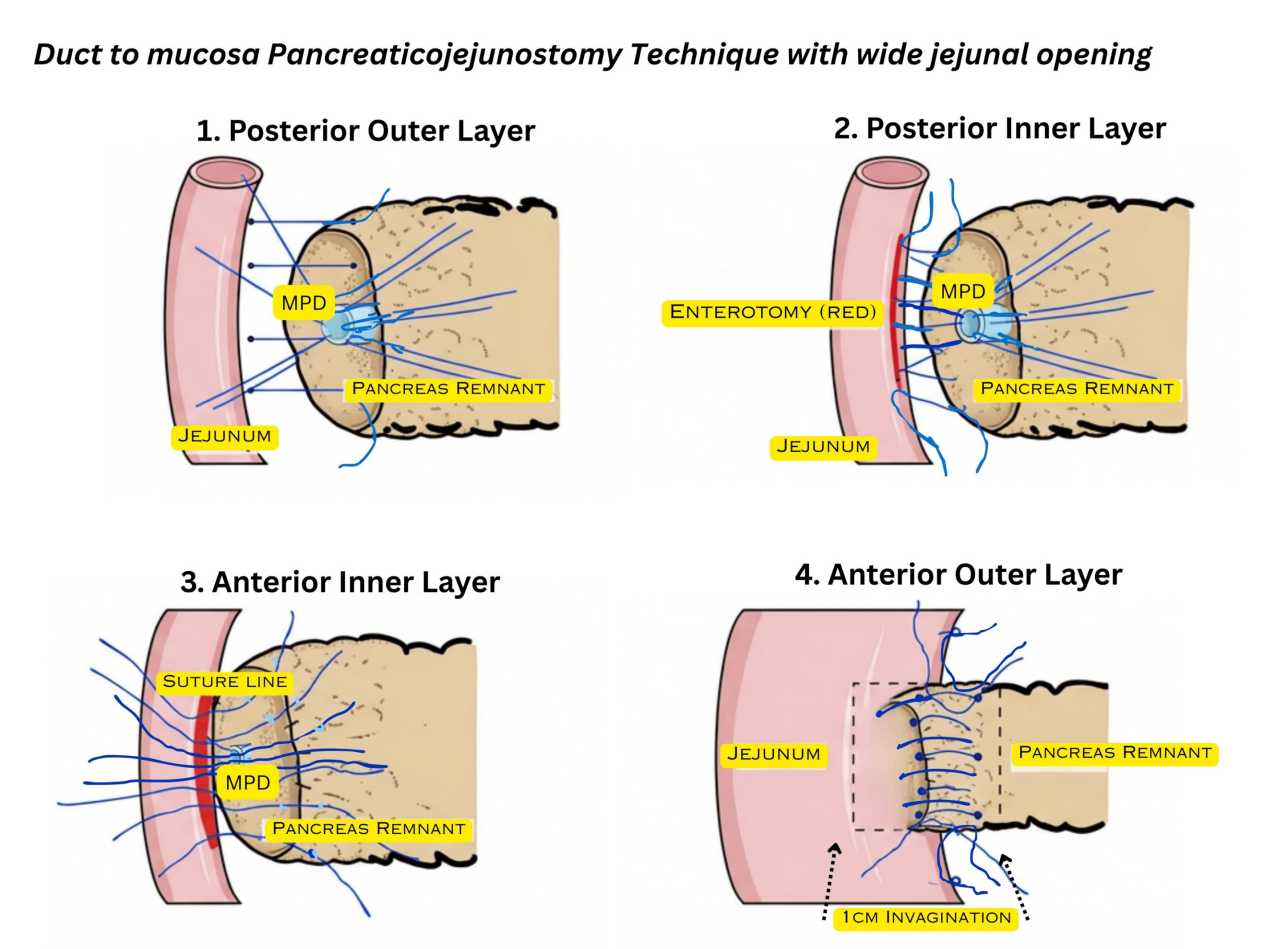
Illustration of duct-to-mucosa pancreaticojejunostomy with a wide jejunal opening. MPD: main pancreatic duct. This figure was created by the author using Canva (Canva Pty Ltd., Sydney, Australia).

Anterior outer layer involves placing sutures between the anterior surface of the pancreas and seromuscular sutures in the jejunum. Tying of these sutures also invaginates the pancreas into the jejunum by 1 cm. Superior and inferior ends are tied with an additional suture (Figure 1D). A schematic diagram illustrating the surgical technique is presented in Figure 2.

The ductal sutures serve to produce adequate duct to mucosa approximation, and seromuscular sutures invaginate the pancreas remnant inside the widely opened jejunum, resulting in a hybrid nature of this PJ. After completion of hepaticojejunostomy and gastrojejunostomy, we routinely place three drains, one in the right subhepatic location, one from the left side in the lesser sac, and one pelvic drain (Video 1).

**Video 1:** Surgical technique of pancreaticojejunostomy with a wide jejunal opening.

## Results

During the study period, 12 consecutive patients underwent PD with double-layer DTM PJ using a wide jejunal opening. Patient demographics and preoperative characteristics are summarized in Table 1. The mean age was 62.33 years (range: 45-90), with male predominance (8/12, 66.7%). Six patients (50%) had PBD for obstructive jaundice. The most common indications for PD were pancreatic head adenocarcinoma and periampullary carcinoma, each 4/12 (33.3%). Three patients (25%) had soft pancreatic texture on intraoperative assessment, and three patients (25%) had a small main pancreatic duct (≤3 mm). The mean pancreaticojejunal anastomosis time was 51.25 minutes (range: 40-60). The median intraoperative blood loss was 550 mL (range: 200-1200 mL).

**Table 1:** Patient characteristics. CBD: common bile duct; PD: pancreaticoduodenectomy; ISGPS: International Study Group of Pancreatic Surgery.

Baseline factor	Frequency
Age (years), mean ± SD (range)	62.33 ± 11.61 (45-90)
Gender	
Male	8 (66.7%)
Female	4 (33.3%)
Diagnosis	
Carcinoma head of the pancreas	4 (33.3%)
Carcinoma uncinate process	3 (25%)
Periampullary carcinoma	4 (33.3%)
Distal CBD carcinoma	1 (8.3%)
Preoperative biliary stent	
No	6 (50%)
Yes	6 (50%)
Anastomosis type	
Pancreaticojejunostomy	12 (100%)
Operative time (minutes), mean ± SD (range)	51.25 ± 7.11 (40-60)
Blood loss (ml)	
≤250	2 (16.7%)
250-500	6 (50%)
500-1000	3 (25%)
>1000	1 (8.3%)
Pancreatic duct size (mm)	
≤3	3 (25%)
>3	9 (75%)
Pancreas texture	
Non-soft	9 (75%)
Soft	3 (25%)
ISGPS classification	
Non-soft and PD ≤ 3 mm	1 (8.3%)
Non-soft and PD > 3 mm	8 (66.7%)
Soft and PD ≤ 3 mm	1 (8.3%)
Soft and PD > 3 mm	2 (16.7%)

No patient (0%, 95% CI: 0-24.2%) developed CR-POPF (grade B or C) as per the ISGPS 2016 criteria. Amylase level at POD three was 20.5 ± 3.63 U/L (range: 13-26) while the average level on POD five was 4.00 ± 2.17U/L (range: 1-8). The levels of amylase in the drained fluid were well below the significant threshold. The overall morbidity rate was 41.7% (5/12 patients) (Table 2). Two patients (16.7%) had minor wound infection (grade I) managed with local care, and two patients (16.7%) had grade II complications. Among them, one had pneumonitis requiring antibiotics, and another had esophageal candidiasis managed with antifungals. One patient (8.3%) had a grade IV complication of pulmonary thromboembolism requiring ICU admission, anticoagulants, and supportive care.

**Table 2:** Postoperative complications.

Postoperative complications	Frequency (%)
Pancreaticojejunostomy leak	
No	12 (100%)
Yes	0 (0%)
Other complications present	
No	7 (58.3%)
Yes	5 (41.7%)
Nature of complication	
Surgical site infection	2 (16.7%)
Thromboembolism	1 (8.3%)
Pneumonitis	1 (8.3%)
Esophageal candidiasis	1 (8.3%)
Complications (Clavien-Dindo grade)	
Grade I	2 (16.7%)
Grade II	2 (16.7%)
Grade IV	1 (8.3%)
Management of complication	
Conservative	5 (41.7%)

Average drain removal time was 13 days in patients who had PBD and 8.6 days in those taken up for surgery directly. Also, there is a tendency toward more extended hospital stay (average 7.6 days versus 7.3 days) and longer time to start oral diet (7.1 days versus 6.3 days) in patients who underwent PBD (Table 3). There was no significant correlation between age and DGE. Those with more severe complications had longer hospital stays. Patients treated for the uncinate process tend to have longer hospital stays compared to other primary sites in this study group.

**Table 3:** Preoperative variables and postoperative outcomes. CBD: common bile duct.

	Mean ± SD	t/F-value	p-value
Preoperative biliary stent * Hospital stay			
No	7.33 ± 1.37	-0.513	0.619
Yes	7.67 ± 0.82		
Delayed gastric emptying (DGE) * Age			
Absent	63.5 ± 9.19	0.149	0.885
Present	62.1 ± 12.45		
Complications (Clavien–Dindo grade) * Hospital stay			
Grade I	7.00 ± 0.00	12.6	0.074
Grade II	7.50 ± 0.71		
Grade IV	10.00 ± 0.00		
Diagnosis * Hospital stay			
Carcinoma of the pancreas head	6.75 ± 0.5	2.736	0.113
Carcinoma uncinate process	8.67 ± 1.15		
Periampullary carcinoma	7.50 ± 1.00		
Distal CBD carcinoma	7.00 ± 0.00		

DGE was a common finding in these patients. Seven patients (58.3%) had grade C, and three (25%) had grade B DGE. Average time to resume full oral diet was 15.92 ± 7.22 (range: 6-25) (Table 4). At 90-day follow-up, no delayed complications were identified.

**Table 4:** Gastrointestinal recovery and follow-up.

Gastrointestinal recovery	Frequency (%)
Delayed gastric emptying (DGE)	
Absent	2 (16.7%)
Present	10 (83.3%)
DGE grade	
Grade B: 7-14 days or reinsertion >7	3 (25%)
Grade C: >14 days or reinsertion > 14	7 (58.3%)
Full oral diet day	15.92 ± 7.22 (range: 6-25)
3-month follow-up	
Morbidity	
No	12 (100%)
Yes	0 (0.0%)

## Discussion

Over time, several techniques have been described for PJ (Table 5). Techniques may differ, but central to the success of all these is appropriate DTM apposition in tension-free, well-vascularized tissue without distal obstruction [22]. The DTM technique is often compared to invagination PJ, which involves invaginating the pancreatic stump into the jejunum [23]. While some studies suggest that invagination may reduce POPF rates in high-risk patients with a soft pancreas, the DTM technique remains widely used due to its simplicity and adaptability to various pancreatic conditions [24]. Blumgart highlights the importance of the DTM anastomosis with a small jejunal opening [25,26]. This technique has been shown to effectively minimize the risk of postoperative complications in both open and minimally invasive techniques [26,27]. Other studies also established the safety of double-layer DTM PJ with a wide jejunal opening [3].

**Table 5:** Techniques of pancreatojejunostomy. CR-POPF: clinically relevant postoperative pancreatic fistula; MPD: main pancreatic duct; POPF: postoperative pancreatic fistula.

Technique	CR-POPF rate	Key advantages
Duct-to-mucosa (DTM)	5%-15%	Precise mucosal apposition, adaptable to small ducts/soft pancreas, low mortality
Invagination	7.7%-10.6%	Reduced fistula rates in soft pancreas cases, shorter hospital stay
Blumgart	4.9%-15.6%	Double-layer suture, small jejunal opening, anchoring of MPD, suitable for all pancreatic textures
Heidelberg technique	6%-12%	Safe technique, minimizes POPF, effective for various pancreatic duct sizes, lower complication rates

The described technique follows the principles of the DTM approach while incorporating small elements of invagination. A wide opening in the jejunum facilitates precise suturing and reduces tension on the anastomosis, particularly important in cases with a small pancreatic duct [3,4]. The use of monofilament sutures has been associated with lower fistula rates and improved healing [6,8]. Placing the knots on the jejunum wall prevents pancreatic parenchymal laceration during tightening [27]. Preoperative evaluation of pancreatic texture and duct size is critical for selecting the optimal anastomotic technique [5,24]. The approach presented here is safe and effective, particularly in patients with challenging pancreatic anatomy. The average duration of time required for this anastomosis is 51.25 ± 7.11 minutes (range: 40-60). In a study comparing the Heidelberg technique of Buchler, which utilizes a wide jejunal incision, with the classical DTM method of Warren and Cattell, there was no statistically significant difference in the POPF rate (10% versus 10%). However, the former technique required less time and was associated with lower morbidity [28]. The Heidelberg technique has also been modified with a small jejunal incision with comparable outcomes [29,30]. The ISGPS also concluded that there is no better way of doing PJ, and the best way to decrease CR-POPF is to follow a standardized method consistently in early carriers, and with experience, surgeons can diversify according to clinical situation [31]. Even further trials are unlikely to establish the superiority of one method over the other [32]. The binding PJ described by Peng et al. showed no PJ fistula, but these results were not reproduced in other reported studies [33,34]. Instead, one study also indicates higher postpancreatectomy hemorrhage [35].

From various trials and consensus statements, at present, the superiority of any single technique is not established. However, what can be concluded safely is that the technique practiced safely and repeatedly in an institution should be standardized consistently [31]. A good level of evidence should back the technical method. The technique used in this study fulfills the basic requirement for performing a safe PJ. In this technique, adequate duct to mucosa approximation is done at the time of securing ductal sutures, and the pancreas is ultimately invaginated by at least 1 cm inside the jejunum by parenchymal and seromuscular sutures. Although the number is small, both soft and non-soft texture pancreas and small and dilated PD were dealt with favorably. In this study, patients with PBD experienced longer hospital stays and extended drain removal times, although POPF was not a concern. Previously published studies also concluded that PBD tends to have higher infection complications and did not find any difference in POPF [36-38]. Although we routinely use an internal PD stent in our cases, stent versus no stent PJ results in similar outcomes [39-41]. As a biochemical measure, we documented drain fluid amylase on POD three and POD five. After that, we remove the drains, one at a time. Hyperamylasemia in the drain fluid helps in the early identification of POPF [42]. In our study, most patients had DGE. It is one of the reasons that we shift our patients to a complete oral diet slowly and remove the enteral feeding tube only after initiation of satisfactory oral feed tolerance. DGE is the most common morbidity after PD and can be observed in 50-60% of patients [43].

Several important limitations must be acknowledged. First, the small sample size (n = 12) severely limits statistical power. Second, the retrospective design introduces potential for bias in data collection and outcome assessment. Third, the absence of a control group precludes direct comparison with alternative techniques. Fourth, single-center and single-surgeon experience raises concerns about external validity.

Despite its limitations, this study has several strengths: (1) consecutive patient series without exclusions, reducing selection bias; (2) standardized surgical technique eliminating technical variability; (3) use of validated ISGPS 2016 criteria for POPF and DGE, facilitating comparison with contemporary literature; and (4) transparent reporting of both favorable (zero POPF) and unfavorable (high DGE) outcomes. This much favorable POPF leak rate at present cannot be endorsed because the numbers are small and inadequate to draw this conclusion. However, it highlights the importance of establishing a standardized technique at the institutional level, and consistent practice and improvement of that safe technique can improve morbidities of PJ. Continuation of this study to include a larger population and further studies are required to draw a better conclusion in the future.

## Conclusions

The double-layer DTM technique with a wide jejunal opening is a safe and effective method of PJ offering low CR-POPF rates and adaptability to various pancreatic conditions. While invagination techniques may have specific advantages, the DTM method remains a cornerstone in pancreatic surgery due to its simplicity, efficacy, and low complication rates. The wide jejunal opening represents an underutilized technical variant that merits rigorous evaluation in larger comparative studies. Such studies should specifically target high-risk patients (soft pancreas, small duct diameter, high fistula risk score) where the potential benefits of improved visualization and reduced anastomotic tension may be most relevant. Future research must also address the high DGE rate through technical refinements and optimized perioperative management protocols.

## References

[1] Manoukian G, Qureshi MR, Bangash A (2026). Management of complications after pancreaticoduodenectomy: a narrative review of pathophysiology and treatment strategies. Cureus.

[2] Gagel AC, Katz MHG (2018). Pancreaticojejunostomy: how I do it. Surgery for Pancreatic and Periampullary Cancer.

[3] Testini M, Piccinni G, Greco L (2011). A modified technique of pancreaticojejunostomy after pancreatoduodenectomy: a preliminary experience. Updates Surg.

[4] Sugiyama M, Suzuki Y, Nakazato T, Yokoyama M, Kogure M, Abe N (2016). Pancreatic duct holder and mucosa squeeze-out technique for duct-to-mucosa pancreatojejunostomy after pancreatoduodenectomy: propensity score matching analysis. World J Surg.

[5] Zhao A, Zhu Q, Qin X (2023). A duct-to-mucosa pancreaticojejunostomy for small main pancreatic duct and soft pancreas in minimally invasive pancreaticoduodenectomy. Surg Endosc.

[6] Zhang B, Li L, Liu H (2023). A modified single-needle continuous suture of duct-to-mucosa pancreaticojejunostomy in pancreaticoduodenectomy. Gland Surg.

[7] Zheng M, Liu A, Li J (2022). Comparison of early postoperative outcomes between omega-like duct-to-mucosa pancreatojejunostomy and conventional duct-to-mucosa pancreatojejunostomy after pancreaticoduodenectomy. HPB (Oxford).

[8] Chiba N, Shimazu M, Okihara M, Sano T, Tomita K, Takano K, Kawachi S (2016). Efficacy of modified technique in pancreatojejunostomy to prevent postoperative pancreatic fistula after pancreatoduodenectomy. Pancreat Disord Ther.

[9] Hao X, Li Y, Liu L, Bai J, Liu J, Jiang C, Zheng L (2024). Is duct-to-mucosa pancreaticojejunostomy necessary after pancreaticoduodenectomy: a meta-analysis of randomized controlled trials. Heliyon.

[10] Senda Y, Shimizu Y, Natsume S (2018). Randomized clinical trial of duct-to-mucosa versus invagination pancreaticojejunostomy after pancreatoduodenectomy. Br J Surg.

[11] Suzuki Y, Fujino Y, Tanioka Y (2002). Selection of pancreaticojejunostomy techniques according to pancreatic texture and duct size. Arch Surg.

[12] Parasyris S, Ntella V, Sidiropoulos T (2024). Modified reconstruction approach after pancreaticoduodenectomy optimizes postoperative outcomes: results from a multivariate cohort analysis. Exp Ther Med.

[13] Büchler MW, Friess H, Wagner M, Kulli C, Wagener V, Z'Graggen K (2000). Pancreatic fistula after pancreatic head resection. Br J Surg.

[14] Z'graggen K, Uhl W, Friess H, Büchler MW (2002). How to do a safe pancreatic anastomosis. J Hepatobiliary Pancreat Surg.

[15] di Mola FF, Grottola T, Panaccio P, Tavano F, De Bonis A, Valvano MR, Di Sebastiano P (2020). End-to-side duct-to-mucosa pancreaticojejunostomy after pancreaticoduodenectomy. A comparison trial of small versus larger jejunal incision. A single center experience. Ann Ital Chir.

[16] Zurleni T, Olmetti S, Marzoli L, Zurleni F (2020). Clinically relevant postoperative pancreatic fistula rates in two methods of pancreatico-jejunal anastomosis compared: evolution of surgical technique in a single centre over time. Res Sq.

[17] Shrikhande SV, Kleeff J, Büchler MW, Friess H (2007). Pancreatic anastomosis after pancreaticoduodenectomy: how we do it. Indian J Surg.

[18] Schuh F, Mihaljevic AL, Probst P (2023). A simple classification of pancreatic duct size and texture predicts postoperative pancreatic fistula: a classification of the International Study Group of Pancreatic Surgery. Ann Surg.

[19] Bassi C, Marchegiani G, Dervenis C (2017). The 2016 update of the International Study Group (ISGPS) definition and grading of postoperative pancreatic fistula: 11 years after. Surgery.

[20] Dindo D, Demartines N, Clavien PA (2004). Classification of surgical complications: a new proposal with evaluation in a cohort of 6336 patients and results of a survey. Ann Surg.

[21] Wente MN, Bassi C, Dervenis C (2007). Delayed gastric emptying (DGE) after pancreatic surgery: a suggested definition by the International Study Group of Pancreatic Surgery (ISGPS). Surgery.

[22] Olakowski M, Grudzińska E, Mrowiec S (2020). Pancreaticojejunostomy—a review of modern techniques. Langenbecks Arch Surg.

[23] Chen HW, Lai EC, Su SY, Cai YF, Zhen ZJ, Lau WY (2008). Modified technique of pancreaticojejunal anastomosis with invagination following pancreaticoduodenectomy: a cohort study. World J Surg.

[24] Zhang S, Lan Z, Zhang J (2017). Duct-to-mucosa versus invagination pancreaticojejunostomy after pancreaticoduodenectomy: a meta-analysis. Oncotarget.

[25] Blumgart LH (1996). A new technique for pancreatojejunostomy. J Am Coll Surg.

[26] Tewari M, Mahendran R, Kiran T, Verma A, Dixit VK, Shukla S, Shukla HS (2019). Outcome of 150 consecutive Blumgart’s pancreaticojejunostomy after pancreaticoduodenectomy. Indian J Surg Oncol.

[27] Long TCD, Dat LT (2022). Modified Blumgart pancretojejunostomy, a simple applicable and safe technique in totally laparoscopic pancreaticoduodenectomy. HPB.

[28] Subhramaniyam S, Kalariya BJ, Guruprasath S, Arul Jothi RDR (2024). Post operative pancreatic fistula rate following pancreaticojejunostomy with Heidelberg technique versus classical duct to mucosa technique: a comparative study. Int Surg J.

[29] Torres OJ, Costa RC, Costa FF, Neiva RF, Suleiman TS, Souza YL, Shrikhande SV (2017). Modified Heidelberg technique for pancreatic anastomosis. Arq Bras Cir Dig.

[30] Chowdappa R, Tiwari AR, Ranganath N, Kumar RV (2019). Modified Heidelberg technique of pancreatic anastomosis postpancreaticoduodenectomy - 10 years of experience. South Asian J Cancer.

[31] Shrikhande SV, Sivasanker M, Vollmer CM (2017). Pancreatic anastomosis after pancreatoduodenectomy: a position statement by the International Study Group of Pancreatic Surgery (ISGPS). Surgery.

[32] Kilambi R, Singh AN (2018). Duct-to-mucosa versus dunking techniques of pancreaticojejunostomy after pancreaticoduodenectomy: Do we need more trials? A systematic review and meta-analysis with trial sequential analysis. J Surg Oncol.

[33] Peng SY, Wang JW, Lau WY, Cai XJ, Mou YP, Liu YB, Li JT (2007). Conventional versus binding pancreaticojejunostomy after pancreaticoduodenectomy: a prospective randomized trial. Ann Surg.

[34] Kim JM, Hong JB, Shin WY, Choe YM, Lee GY, Ahn SI (2014). Preliminary results of binding pancreaticojejunostomy. Korean J Hepatobiliary Pancreat Surg.

[35] Maggiori L, Sauvanet A, Nagarajan G, Dokmak S, Aussilhou B, Belghiti J (2010). Binding versus conventional pancreaticojejunostomy after pancreaticoduodenectomy: a case-matched study. J Gastrointest Surg.

[36] Gong L, Huang X, Wang L, Xiang C (2020). The effect of preoperative biliary stents on outcomes after pancreaticoduodenectomy: a meta-analysis. Medicine (Baltimore).

[37] Garcia-Ochoa C, McArthur E, Skaro A, Leslie K, Hawel J (2021). Pre-operative stenting and complications following pancreatoduodenectomy for pancreatic cancer: an analysis of the ACS-NSQIP registry. Surg Endosc.

[38] Pisters PW, Hudec WA, Hess KR (2001). Effect of preoperative biliary decompression on pancreaticoduodenectomy-associated morbidity in 300 consecutive patients. Ann Surg.

[39] Hong S, Wang H, Yang S, Yang K (2013). External stent versus no stent for pancreaticojejunostomy: a meta-analysis of randomized controlled trials. J Gastrointest Surg.

[40] Mazzola M, Zironda A, Giani A (2025). Biodegradable internal stent versus no stent for patients at increased risk of pancreatic fistula after pancreaticoduodenectomy: a single-center propensity score matching analysis. Updates Surg.

[41] Farooqui W, Tschuor C, Storkholm JH, Krohn PS, Hansen CP, Burgdorf SK (2025). Impact of biodegradable stent on pancreatic leakage after pancreatoduodenectomy - systematic review. Ann Med Surg (Lond).

[42] Bannone E, Marchegiani G, Vollmer C (2023). Postoperative serum hyperamylasemia adds sequential value to the fistula risk score in predicting pancreatic fistula after pancreatoduodenectomy. Ann Surg.

[43] Busquets J, Martín S, Secanella L (2022). Delayed gastric emptying after classical Whipple or pylorus-preserving pancreatoduodenectomy: a randomized clinical trial (QUANUPAD). Langenbecks Arch Surg.

